# Partial Nephrectomy Versus Radical Nephrectomy for Endophytic Renal Tumors: Comparison of Operative, Functional, and Oncological Outcomes by Propensity Score Matching Analysis

**DOI:** 10.3389/fonc.2022.916018

**Published:** 2022-07-26

**Authors:** Situ Xiong, Ming Jiang, Yi Jiang, Bing Hu, Ru Chen, Zhijun Yao, Wen Deng, Xianwen Wan, Xiaoqiang Liu, Luyao Chen, Bin Fu

**Affiliations:** ^1^ Department of Urology, The First Affiliated Hospital of Nanchang University, Nanchang, China; ^2^ Jiangxi Institute of Urology, Nanchang, China; ^3^ Department of Urology, The Second Affiliated Hospital of Nanchang University, Nanchang, China; ^4^ Department of Anesthesiology, The First Affiliated Hospital of Nanchang University, Nanchang, China

**Keywords:** endophytic renal tumor, partial nephrectomy, radical nephrectomy, propensity score matching, oncological outcomes, function outcomes, operative outcomes

## Abstract

**Purpose:**

The study aimed to compare operative, functional, and oncological outcomes between partial nephrectomy (PN) and radical nephrectomy (RN) for entophytic renal tumors (ERTs) by propensity score matching (PSM) analysis.

**Methods:**

A total of 228 patients with ERTs who underwent PN or RN between August 2014 and December 2021 were assessed. A PSM in a 1:1 ratio was conducted to balance the differences between groups. Perioperative characteristics, renal functional, and oncological outcomes were compared between groups. Univariate and multivariate logistic and Cox proportional hazard regression analyses were used to determine the predictors of functional and survival outcomes.

**Results:**

After PSM, 136 cases were matched to the PN group (n = 68) and the RN group (n = 68). Patients who underwent RN had shorter OT, less EBL, and lower high-grade complications (all *p* <0.05) relative to those who underwent PN. However, better perseveration of renal function was observed in the PN group, which was reflected in 48-h postoperative AKI (44.1% vs. 70.6%, *p* = 0.002), 1-year postoperative 90% eGFR preservation (45.6% vs. 22.1%, *p* = 0.004), and new-onset CKD Stage ≥III at last follow-up (2.9% vs. 29.4%, *p* <0.001). RN was the independent factor of short-term (OR, 2.812; 95% CI, 1.369–5.778; *p* = 0.005) and long-term renal function decline (OR, 10.242; 95% CI, 2.175–48.240; *p* = 0.003). Furthermore, PN resulted in a better OS and similar PFS and CSS as compared to RN (*p* = 0.042, 0.15, and 0.21, respectively). RN (OR, 7.361; 95% CI, 1.143–47.423; *p* = 0.036) and pT3 stage (OR, 4.241; 95% CI, 1.079–16.664; *p* = 0.039) were independent predictors of overall mortality.

**Conclusion:**

Among patients with ERTs, although the PN group showed a higher incidence of high-grade complications than RN, when technically feasible and with experienced surgeons, PN is recommended for better preservation of renal function, longer OS, and similar oncological outcomes.

## Introduction

Endophytic renal tumors (ERTs) are tumors surrounded by normal renal parenchyma and attributed to three points of the E-element in the R.E.N.A.L. Nephrometry Score (RENAL-NS) system (1–3). Most ERTs are small spherical masses in deep locations and do not protrude from the renal surface of the tumor. Partial nephrectomy (PN) is the accepted standard treatment for normal small renal masses (4), with superior long-term benefit (5, 6).

Due to its highly complex anatomy, it is difficult to remove tumors and suture incised renal parenchyma, which requires considerable expertise and higher technical skills. Furthermore, these cases are related to higher intraoperative and perioperative complication rates, including positive resection margin caused by an unclear boundary, massive bleeding due to an accidental vascular injury, urine leakage caused by an accidental pelvicalyceal system injury, and renal vascular occlusion caused by inappropriate suture (7–9). Given the above risks and challenges, in the past, most urologists preferred RN for ERMs to avoid serious complications (10, 11). Owing to technological and conceptual advances, some authors have reported the successful application of PN for ERTs (9, 10, 12–14). These results demonstrate PN as a feasible technique for such anatomically complicated renal tumors. However, given technical difficulties due to this procedure, limited evidence of oncological and functional outcomes is available. Thus, it is unknown whether PN is more appropriate for ERTs than RN.

Thus far, no report of comparison between PN and RN for the treatment of ERTs has been published. We aimed to compare the operative, functional, and oncological outcomes for ERTs by PN or RN treatment by propensity score-matching (PSM) analysis; these findings may guide the treatment of ERTs.

## Materials and Methods

### Patient Cohort

**Figure 1:**
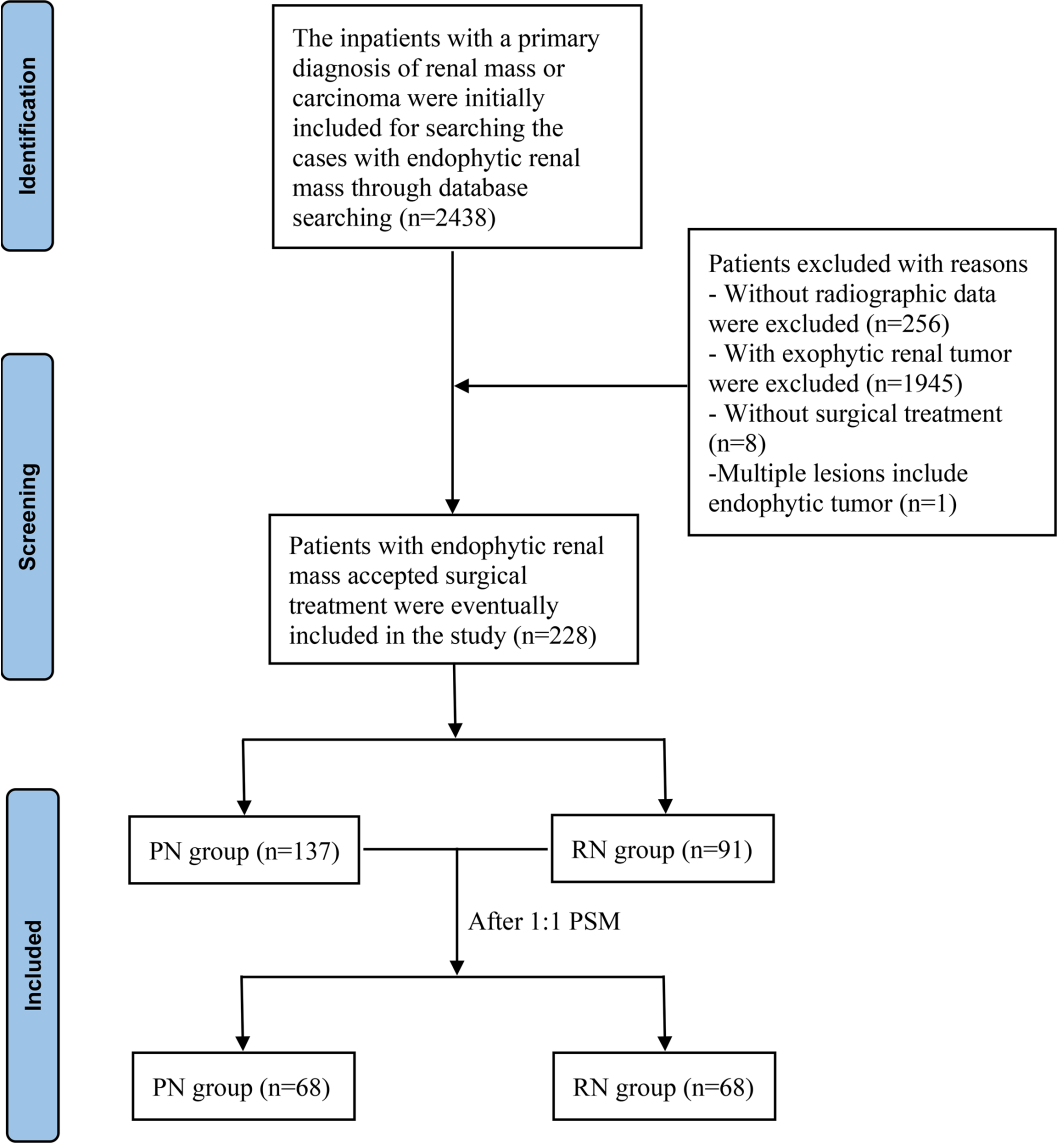
Flow chart for inclusion of patients. PN, partial nephrectomy; RN, radical nephrectomy.

Patients with ERTs who underwent PN or RN between 1 August 2014 and 31 December 2021 at the First Affiliated Hospital of Nanchang University were assessed retrospectively. Of the 2,438 patients with a primary diagnosis of renal tumor or carcinoma, 228 patients with ERTs were identified and included in this study based on the following inclusion criteria: (1) imaging assessment of the location of the tumor was preserved in our radiographic database; (2) ERTs that were surrounded by normal renal parenchyma and attributed to three points for the E-element in the RENAL-NS system (3); (3) those who accepted surgical treatment by PN or RN, and (4) those without multiple lesions included endophytic masses (n >2), including multiple renal angiomyolipomas with endophytic lesion. The flowchart for the enrollment of patients with ERTs is shown in **Figure 1**. Patients who simultaneously met the above inclusion criteria (n = 228) were divided into the PN (n = 131) and RN (n = 97) groups according to the surgical method.

All operations were conducted by highly experienced urologists using laparoscopic or robot-assisted laparoscopic techniques; no open surgery was performed. After adequate exposure to the kidney and renal artery, intraoperative ultrasound was performed to locate the renal tumor during PN. The proximal renal artery away from the tumor was then clamped with bull’s-head forceps to separate the tumor from the normal renal tissue with a pair of cold scissors, and two layers of suturing were performed to close the renal injury. Afterward, the bulldog forceps were removed for hemostatic evaluation, and the warm ischemia time was recorded. Apart from intraoperative ultrasound to locate tumors during PN, other detailed surgical procedures of PN and RN through laparoscopic or robot-assisted laparoscopic techniques for ERTs were the same as those for exophytic tumors described previously (14–16).

### Research Materials

The demographic characteristics, including age, gender, body mass index (BMI), diabetes mellitus (DM), hypertension (HTN), chronic kidney disease (CKD) Stage ≥III, abdominal surgery history, age-adjusted Charlson’s comorbidity index (ACCI), Eastern Cooperative Oncology Group Performance Status (ECOG PS), American Society of Anesthesiologists (ASA), preoperative serum creatinine (Scr), estimated glomerular filtration rates (eGFR), preoperative hemoglobin, and surgical technique were extracted from the prospectively managed clinical database. The Cockcroft–Gault (C–G) formula was used to calculate eGFR, and all values were standardized by body surface area of patients.

The oncology characteristics, including tumor size, location, laterality, clinical T stage (cTn), and RENAL-NS, were assessed by reviewing the computed tomography (CT) or magnetic resonance imaging (MRI) from our radiographic database. All the imaging information was blindly assessed and collected by urologists SX and Mj, and disputed cases were evaluated by a more senior urologist, LC.

Collected perioperative outcomes included operating time (OT), warm ischemia time (WIT), estimated blood loss (EBL), perioperative transfusion rate, surgery conversion rate, restoration time of bowel functions, drainage tube removal time, postoperative hospitalization time, postoperative complications, 48-h postoperative Scr, 48-h postoperative eGFR, 48-h postoperative eGFR descent, and 48-h postoperative acute kidney injury (AKI). Postoperative complications were determined by the Clavien–Dindo classification (17). AKI was defined as either a >50% increase in the postoperative serum creatinine relative to preoperative Scr or an absolute increase of >0.3 mg/dl according to the Acute Kidney Injury Network (AKIN) criteria (18).

All tumor specimens were reviewed for diagnosis by a single urological pathologist. The pathologic characteristics consisted of histological subtype, TNM stage, Fuhrman nuclear grade (I/II grade were classified into low grade and III/IV into high), parasitic (or tentacular) invasion, and distant extension according to the eighth edition of the American Joint Committee on Cancer (AJCC) cancer staging manual.

According to the standardized institutional postoperative protocol, the regular follow-up was conducted every 3 months after surgery for 6 months, every 6 months for the next 3 years, and annually after that. The essential oncology follow-up consisted of CT or MRI scans for the chest and entire abdomen. The changes in eGFR calculated using Scr followed up at postoperative 3-, 6-, and 12 months and 3- and 5 years reflected the renal function outcomes. Survival outcomes included cancer-specific survival (CSS), progression-free survival (PFS), and overall survival (OS), defined as the interval from the date of surgery to death related to the renal tumor; the first tumor recurrence, or metastasis, and death due to any cause, respectively.

### Statistical Analysis

To eliminate differences between groups in preoperative demographic and tumor characteristics, PSM analysis was performed using variables of age, gender, BMI, DM, HTN, CKD, abdominal surgery history, ACCI, ECOG PG, ASA, preoperative Scr, preoperative eGFR, preoperative hemoglobin, tumor laterality, tumor size, clinical T stage, RENAL-NS, and surgical technique. The propensity score was evaluated by non-parsimonious multivariate logistic regression. Finally, 68 patients in the PN group were successfully matched with a nearest neighbor matching algorithm to the same number of patients in the RN group at a 1:1 ratio. The preoperative covariate differences between the two groups before and after PSM were compared.

All categorical variables compared using the Pearson χ^2^ test were presented in the form of numbers and percentages. As for continuous variables, the normally distributed variables using the Student’s t-test are presented as mean and standard deviation, and the non-normally distributed variables using the Wilcoxon rank sum test are presented as median and interquartile range. A Kaplan–Meier survival analysis was used to compare OS, PFS, and CSS outcomes between groups. Univariate and multivariate logistics and Cox proportional hazard regression were used to determine the predictors of functional and survival outcomes. The univariate results were used to determine the candidate variables for the final multivariate model. All statistical analyses were performed using SPSS 24.0 software (SPSS, Chicago, IL, USA) and R software (version 4.1.0). The *p* <0.5 level was considered statistically significant.

## Results

During the study period, 228 patients with ERTs were included in the final analysis based on the inclusion and exclusion criteria. A total of 131 and 97 patients underwent PN and RN, respectively. According to the demographic and clinical characteristics shown in **Table 1**, statistically, significant differences are found for some variables before PSM. The patients in the RN group showed higher mean age (51.4 vs. 47.5 years, *p* = 0.037), lower mean BMI (22.9 vs. 23.7, *p* = 0.039), bigger tumor size (4.0 vs. 2.8 cm, *p* <0.001), higher overall RENAL-NS (10.1 vs. 8.8, *p* <0.001), higher R score (1.5 vs. 1.2, *p* <0.001), higher N score (3.0 vs. 2.5, *p* <0.001), and higher L score (2.6 vs. 2.2, *p* <0.001) relative to the PN group. Furthermore, the RN group had a higher ratio of ECOG PS score of ≥2 (13.4% vs. 5.3, *p* = 0.033), clinical T stage ≥cT1b (49.5% vs. 15.3%, *p* <0.001), hilar location (43.3% vs. 15.3%, *p* <0.001), and a lower ratio of robot-assisted laparoscopic technique (18.6% vs. 37.4%, *p* = 0.002) compared to the PN group. No statistical difference was found in other variables between groups was observed. After performing 1:1 PSM, all statistically significant covariates achieved a good balance among the matched groups (with 68 patients in both PN and RN groups). The median follow-up duration in the PN and RN groups before the PSM was 41.2 and 45.3 months, respectively; after the PSM, the corresponding values were 44.8 and 45.2 months, respectively.

**Table 1:** Preoperative basic characteristics of patients before and after propensity score matching.

Variables	Before propensity score matching			After propensity score matching		
	PN (n = 131)	RN (n = 97)	*p*-value	PN (n = 68)	RN (n = 68)	*p*-value
Age, years, mean (SD)	47.5 (12.7)	51.4 (15.3)	**0.037**	46.8 (13.7)	49.7 (14.8)	0.230
Gender (male), n (%)	75 (57.3%)	56 (57.7%)	0.942	35 (51.5%)	38 (55.9%)	0.606
BMI, mean (SD)	23.7 (2.8)	22.9 (2.9)	**0.039**	23.4 (2.8)	23.1 (2.9)	0.479
Diabetes mellitus, n (%)	12 (9.2%)	7 (7.2%)	0.529	7 (10.3%)	4 (5.9%)	0.345
Hypertension, n (%)	21 (16.0%)	17 (17.5%)	0.887	10 (14.7%)	9 (13.2%)	0.805
CKD Stage ≥III, n (%)	13 (9.9%)	12 (12.4%)	0.559	6 (8.8%)	7 (10.3%)	0.771
Abdominal surgery history, n (%)	23 (17.6%)	14 (14.4%)	0.527	10 (14.7%)	10 (14.7%)	NA
ACCI score, mean (SD)	4.1 (1.7)	4.3 (1.9)	0.265	4.0 (1.8)	4.2 (1.8)	0.534
ECOG PS score ≥2, n (%)	7 (5.3%)	13 (13.4%)	**0.033**	4 (5.8%)	6 (8.8%)	0.511
ASA score ≥2, n (%)	73 (55.7%)	53 (53.6%)	0.870	41 (60.3%)	37 (54.4%)	0.488
Scr, mg/dl, mean (SD)	0.88 (0.33)	0.86 (0.47)	0.726	0.83 (0.25)	0.82 (0.31)	0.791
eGFR, ml/min/1.73 m^2^, mean **(**SD**)**	91.5 (26.4)	89.0 (26.7)	0.480	94.8 (26.9)	92.7 (26.8)	0.644
Hemoglobin, g/dl, mean (SD)	132.8 (15.6)	129.8 (16.4)	0.221	130.7 (16.6)	130.0 (14.8)	0.802
Laterality (left), n (%)	72 (55.0%)	48 (49.5%)	0.413	37 (54.4%)	36 (52.9%)	0.863
Tumor size, cm, mean (SD)	2.8 (1.0)	4.0 (1.5)	**<0.001**	3.3 (1.0)	3.6 (1.3)	0.077
Clinical T stage ≥cT1b, n (%)	20 (15.3%)	48 (49.5%)	**<0.001**	20 (29.2%)	28 (41.2%)	0.151
RENAL-NS, mean (SD)	8.8 (1.5)	10.1 (1.2)	**<0.001**	9.4 (1.3)	9.8 (1.2)	0.065
R score, mean (SD)	1.2 (0.4)	1.5 (0.6)	**<0.001**	1.3 (0.5)	1.4 (0.5)	0.149
N score, mean (SD)	2.5 (0.8)	3.0 (0.3)	**<0.001**	2.8 (0.6)	2.9 (0.4)	0.067
L score, mean (SD)	2.2 (0.9)	2.6 (0.7)	**<0.001**	2.4 (0.9)	2.5 (0.8)	0.309
Hilar location, n (%)	20 (15.3%)	42 (43.3%)	**<0.001**	17 (25.0%)	24 (35.3%)	0.191
Surgical Technique			**0.002**			0.252
Open	0	0		0	0	
Laparoscopic, n (%)	82 (62.6%)	79 (81.4%)		46 (67.6%)	52 (76.5%)	
Robot, n (%)	49 (37.4%)	18 (18.6%)		22 (32.4%)	16 (23.5%)	

RN, radical nephrectomy; PN, partial nephrectomy; SD, standard deviation; BMI, body mass index; CKD, chronic kidney disease, CKD Stage ≥III defined as eGFR<60 ml/min; Sc, serum creatinine; eGFR, estimated glomerular filtration rate; ACCI, age-adjusted Charlson's comorbidity index; ECOG PS, Eastern Cooperative Oncology Group Performance Status; ASA, American Society of Anesthesiologists; RENAL-NS, R.E.N.A.L. Nephrometry Score.

The bold numbers mean statistically difference.

Operative, pathological, and renal functional outcomes for PN and RN groups after PSM are shown in **Table 2**. Patients in the PN group showed longer OT (194.4 vs. 171.3 mins, *p* = 0.014) and more EBL (198.5 vs. 140.7 ml, *p* = 0.038) than those in the RN group, whereas no statistical difference in required blood transfusion (2.9% vs. 5.9%, *p* = 0.680) was observed. Postoperative recovery indices, which included restoration time of bowel functions, drainage tube removal time, and postoperative hospitalization time, were similar between the groups. Two cases of surgical conversion (to RN) for intraoperative massive and repeated bleeding in the PN group, and no conversion in the RN group, or to open method were recorded. The mean warm ischemia time in the PN group was 27.6 min. No positive resection margins were observed in either group. Patients in the PN group had a similar ratio of overall and low-grade (Clavien–Dindo grades I–II) complications (*p* = 0.060 and *p* = 0.341, respectively) but showed a higher ratio of high-grade (Clavien–Dindo grades III–IV) complications (10.3% vs. 1.5%, *p* = 0.029) relative to the RN group. High-grade complications included ICU management (n = 1), urine leak (n = 3), hemorrhage treated by embolization (n = 1), acute renal failure (n = 1), and the second operation for suspected residual tumor (n = 1) in the PN group, and acute renal failure (n = 1) in the RN group.

**Table 2:** Perioperative and oncological outcomes for PN and RN after propensity score matching.

Variables	PN (n = 68)	RN (n = 68)	*p*-value
OT, min, mean (SD)	194.4 (48.8)	171.3 (58.6)	**0.014**
WIT, min, median (IQR)	27.5 (26.3-29.0)	–	–
EBL, mL, median (IQR)	200 (100-200)	100 (100-150)	**<0.001**
Transfusion, n (%)	2 (2.9%)	4 (5.9%)	0.680
Surgery conversion, n (%)			
To RN	2 (2.9%)	–	–
Positive resection margin, n (%)	0	0	–
Restoration time of bowel functions, days, median (IQR)	2 (2-3)	2 (2-3)	0.305
Drainage tube removal time, days, median (IQR)	3 (3-5.75)	3 (3-4)	0.804
Postoperative hospitalization time, days, median (IQR)	7 (5-9)	6 (5-8)	0.061
Postoperative complications, n (%)	19 (27.9%)	10 (14.7%)	0.060
Clavien–Dindo grades I–II, n (%)	12 (17.6%)	9 (13.2%)	0.341
Clavien–Dindo grades III–IV, n (%)	7 (10.3%)	1 (1.5%)	**0.029**
ICU management	1 (1.5%)	0	–
Urine leak	3 (4.4%)	0	–
Hemorrhage treated by embolization	1 (1.5%)	0	–
Acute renal failure	1 (1.5%)	1 (1.5%)	–
Second operation	1 (1.5%)	0	–
Histologic subtype			0.062
Benign, n (%)	15 (22.1%)	7 (10.3%)	
Malignant, n (%)	53 (77.9%)	61 (89.7%)	
ccRCC, n (%)	46 (86.8%)	47 (77.1%)	–
pRCC, n (%)	2 (3.8%)	6 (9.8%)	–
chRCC, n (%)	1 (1.9%)	3 (4.9%)	–
Others, n (%)	4 (7.5%)	5 (8.2%)	–
Pathologic stage			0.115
pT1, n (%)	50 (94.3%)	52 (85.2%)	
pT3, n (%)	3 (5.7%)	9 (14.8%)	
Fuhrman grade			0.247
Low grade (I/II), n (%)	44 (91.7%)	42 (84.0%)	
High grade (III/IV), n (%)	4 (5.8%)	8 (16.0%)	
Lymph node metastasis, n (%)	0	2 (2.9%)	–

RN, radical nephrectomy; PN, partial nephrectomy; OT, operating time; WIT, warm ischemia time; EBL, estimated blood loss; SD, standard deviation; ICU, intensive care unit; ccRCC, clear cell renal cell carcinoma; pRCC, papillary renal cell carcinoma; chRCC, chromophobe renal cell carcinoma; eGFR, estimated glomerular filtration rate.

The bold numbers mean statistically difference.

Among pathologic characteristics, no statistically significant differences in histological subtype, pathological stage, or Fuhrman grade were observed between the two groups (all *p* >0.05).

**Figure 2:**
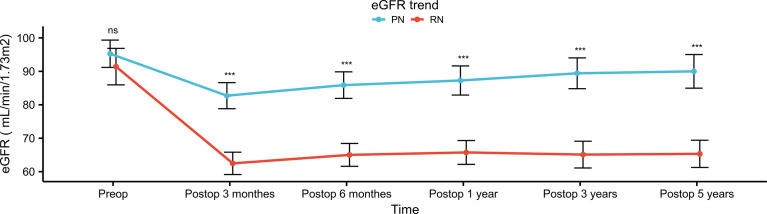
Postoperative eGFR trend of patients in PN group and RN group after propensity score matching. eGFR, estimated glomerular filtration rate; PN, partial nephrectomy; RN, radical nephrectomy. "***" means p<0.001; "ns" means not statistically significant.

Among postoperative renal functional outcomes (**Table 3**), the PN group showed a significant association with higher 48-h postoperative eGFR (70.5 vs. 57.4 ml/min/1.73m^2^, *p* <0.001) than the RN group. Compared with preoperative eGFR, 48-h postoperative eGFR decreased in the PN and RN groups by 24.3 and 35.3 ml/min/1.73m^2^, respectively (*p* = 0.002). Univariate and multivariate logistic analyses showed that RN (OR, 2.812; 95% CI, 1.369–5.778; *p* = 0.005) was an independent risk factor for 48-h postoperative AKI (**Table 4**). Throughout the follow-up period, the renal function in the PN group was significantly better than that in the RN group (all *p* <0.001) (**Figure 2**). One-year postoperative 90% eGFR preservation occurred in 45.6% of patients in the PN group and 22.1% in the RN group (*p* = 0.004). Furthermore, the patients in the PN group had a lower rate of new-onset CKD Stage ≥III at the last follow-up relative to the RN group (2.9% vs. 29.4%, *p* <0.001). The results of univariate and multivariate logistic analyses suggested that hilar location (OR, 3.726; 95% CI, 1.283–10.823; *p* = 0.016) and RN (OR, 10.242; 95% CI, 2.175–48.240; *p* = 0.003) were independent risk factors for new-onset CKD Stage ≥III at the last follow-up (**Table 5**).

**Table 3:** Preoperative functional outcomes for PN and RN after propensity score matching.

Variables	PN (n = 68)	RN (n = 68)	*p*-value
Preoperative Scr, mg/dl, mean (SD)	0.83 (0.25)	0.82 (0.31)	0.791
Preoperative eGFR, ml/min/1.73 m^2^, mean (SD)	94.8 (26.9)	92.7 (26.8)	0.644
48-h postoperative Scr, mg/dl, mean (SD)	1.13 (0.42)	1.24 (0.47)	0.157
48-h postoperative eGFR, ml/min/1.73 m^2^, mean (SD)	70.5 (22.8)	57.4 (17.5)	**<0.001**
48-h postoperative eGFR descend, ml/min/1.73 m^2^, mean (SD)	23.9 (16.6)	35.3 (23.8)	**0.001**
48-h postoperative AKI, n (%)	30 (44.1%)	48 (70.6%)	**0.002**
1-year postoperative Scr, mg/dl, mean (SD)	0.98 (0.40)	1.19 (0.59)	**0.021**
1-year postoperative eGFR, ml/min/1.73 m^2^, mean (SD)	84.4 (30.1)	66.7 (21.6)	**<0.001**
1-year postoperative 90% eGFR preservation, n (%)	31 (45.6%)	15 (22.1%)	**0.004**
Last follow-up Scr, mg/dl, mean (SD)	0.99 (0.43)	1.22 (0.74)	**0.028**
Last follow-up eGFR, ml/min/1.73 m^2^, mean (SD)	82.1 (24.9)	66.1 (19.4)	**<0.001**
New-onset CKD Stage ≥III at last follow-up, n (%)	2 (2.9%)	20 (29.4%)	**<0.001**

RN, radical nephrectomy; PN, partial nephrectomy; SD, standard deviation; Scr, serum creatinine; eGFR, estimated glomerular filtration rate; AKI, acute kidney injury; CKD, chronic kidney disease.

The bold numbers mean statistically difference.

**Table 4:** Univariate and multivariate Logistic analysis of independent risk factors for 48-h postoperative AKI.

Variables	Univariate Analysis		Multivariate Analysis	
	Crude OR (95% CI)	*p*-value	Adjusted OR (95% CI)	*p*-value
Age	1.01 (0.99–1.04)	0.260		
Gender				
Female (Re.) vs. male	0.90 (0.46–1.78)	0.763		
BMI	1.11 (0.98–1.26)	0.109		
Diabetes mellitus	0.59 (0.17–2.05)	0.409		
Hypertension	1.03 (0.38–2.74)	0.959		
Abdominal surgery history	0.71 (0.27–1.83)	0.473		
ACCI score	0.98 (0.81–1.19)	0.844		
ECOG PS score ≥2	0.73 (0.20–2.64)	0.626		
ASA score ≥2	0.81(0.41–1.61)	0.543		
Preoperative eGFR	1.01 (1.00–1.02)	0.201		
Preoperative hemoglobin	0.99 (0.96–1.01)	0.185		
Laterality				
Left (Re.) vs. Right	1.81 (0.91–3.62)	0.092		
Tumor size	0.98 (0.73–1.30)	0.865		
RENAL-NS	1.03 (0.80–1.34)	0.807		
R score	1.24 (0.60–2.57)	0.554		
N score	1.13 (0.58–2.17)	0.725		
L score	0.95 (0.63–1.42)	0.793		
Hilar location	0.81 (0.39–1.69)	0.567		
Technique				
Laparoscopic (Re.) vs. Robot	0.97 (0.45–2.07)	0.937		
Operating method				
PN (Re.) vs. RN	3.04 (1.50–6.17)	**0.002**	2.81 (1.37–5.78)	**0.005**
Operating time	1.00 (0.99–1.00)	0.154		
Estimated blood loss	1.00 (0.99–1.00)	0.199		
Transfusion	1.51 (0.27–8.56)	0.639		
Postoperative hospitalization time	0.85 (0.74–0.98)	**0.024**	0.87 (0.76–1.00)	0.055
Postoperative complications	1.96 (0.79–4.86)	0.144		
Histologic subtype				
Benign (Re.) vs. Malignant	1.77 (0.71–4.45)	0.221		
Pathologic stage				
pT1 (Re.) vs. pT3	0.88 (0.26–3.05)	0.844		
Fuhrman grade				
I/II (Re.) vs. III–IV	0.90 (0.26–3.06)	0.864		

BMI, body mass index; ACCI, age-adjusted Charlson's comorbidity index; ECOG PS, Eastern Cooperative Oncology Group Performance Status; ASA, American Society of Anesthesiologists; eGFR, estimated glomerular filtration rate; Re, reference; RENAL-NS, RENAL-Nephrometry Score; RN, radical nephrectomy; PN, partial nephrectomy.

The bold numbers mean statistically difference.

**Table 5:** Univariate and multivariate Logistic analysis of independent risk factors for new onset CKD stage ≥ II at last follow-up.

Variables	Univariate Analysis		Multivariate Analysis	
	Crude OR (95% CI)	*p*-value	Adjusted OR (95% CI)	*p*-value
Age	1.04 (1.00–1.08)	**0.030**	1.04 (1.00–1.08)	0.060
Gender				
Female (Re.) vs. male	0.68 (0.27–1.69)	0.675		
BMI	0.88 (0.79–1.03)	0.110		
Diabetes mellitus	0.50 (0.06–4.08)	0.495		
Hypertension	1.47 (0.44–4.93)	0.536		
Abdominal surgery history	1.36 (0.41–4.54)	0.616		
ACCI score	1.14 (0.89–1.45)	0.305		
ECOG PS score ≥2	1.23 (0.67–2.27)	0.511		
ASA score ≥2	1.09 (0.43–2.75)	0.857		
Preoperative eGFR	0.99 (0.97–1.01)	0.361		
Preoperative hemoglobin	0.99 (0.97–1.02)	0.697		
Laterality				
Left (Re.) vs. Right	2.32 (0.90–5.97)	0.081		
Tumor size	0.99 (0.67–1.46)	0.961		
RENAL-NS	0.95 (0.67–1.34)	0.775		
R score	1.14 (0.44–2.96)	0.783		
N score	0.89 (0.39–2.06)	0.785		
L score	0.90 (0.53–1.53)	0.701		
Hilar location	3.52 (1.38–9.00)	**0.009**	3.73 (1.28–10.82)	**0.016**
Technique				
Laparoscopic (Re.) vs Robot	1.04 (0.37–2.90)	0.939		
Operating method				
PN (Re.) vs RN	13.8 (3.07–61.65)	**0.001**	10.24 (2.18–48.24)	**0.003**
Postoperative complications	1.35 (0.36–4.99)	0.655		
48-h postoperative AKI	2.95 (1.02–8.55)	**0.046**	1.79 (0.54–5.87)	0.339
Histologic subtype				
Benign (Re.) vs Malignant	1.27 (0.34–4.77)	0.724		
Pathologic stage				
pT1 (Re.) vs pT3	2.09 (0.51–8.60)	0.306		
Fuhrman grade				
I/II (Re.) vs III–IV	1.74 (0.42–7.24)	0.447		

BMI, body mass index; ACCI, age-adjusted Charlson's comorbidity index; ECOG PS, Eastern Cooperative Oncology Group Performance Status; ASA, American Society of Anesthesiologists; eGFR, estimated glomerular filtration rate; RENAL-NS, RENAL-Nephrometry Score; RN: radical nephrectomy; PN, partial nephrectomy.

The bold numbers mean statistically difference.

**Figure 3:**
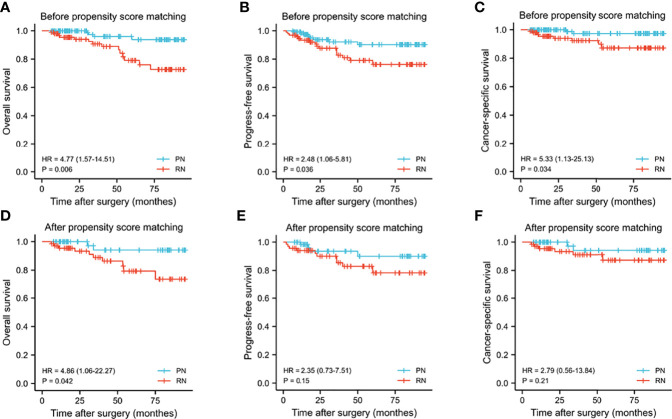
Kaplan–Meier curves for overall survival **(A, D)**, progress-free survival **(B, E)**, cancer-specific survival **(C, F)** between the PN and RN groups before and after propensity score matching. PN, partial nephrectomy; RN, radical nephrectomy.

During the follow-up in the matched cohort, 8 and 16 patients developed local recurrence/distant metastasis in the PN and RN groups, respectively. The overall mortality was 4 patients in the PN group and 15 in the RN group. Cancer-related mortality occurred in 2 patients in the PN group and 8 in the RN group. Kaplan–Meier analyses suggested statistically significant differences in CSS, PFS, and OS in favor of PN (*p* = 0.006, 0.036, and 0.034, respectively) (**Figure 3**). Within the matched cohort, the patients in the PN group showed a longer OS compared with those in the RN group (*p* = 0.042). However, no statistically significant differences were observed in CSS and FPS between the two groups (*p* = 0.15 and 0.21, respectively). Univariate and multivariate Cox regression analyses showed that RN (OR, 7.36; 95% CI, 1.14–47.42; *p* = 0.036) and pathological T3 (pT3) stages were the predictors of overall mortality (OR, 4.241; 95% CI, 1.079–16.664; *p* = 0.039) (**Table 6**).

**Table 6:** Univariate and multivariate Cox analysis of independent risk factors for overall mortality.

Variables	Univariate Analysis		Multivariate Analysis	
	Crude OR (95% CI)	*p*-value	Adjusted OR (95% CI)	*-*value
Age	1.11 (1.05–1.17)	**<0.001**	1.07 (0.95–1.20)	0.285
Gender				
Female (Re.) vs. male	1.89 (0.51–7.00)	0. 343		
BMI	1.04 (0.84–1.30)	0.725		
Diabetes mellitus	6.09 (1.63–22.70)	**0.007**	7.19 (0.98–36.45)	0.052
Hypertension	2.98 (0.79–11.30)	0.108		
Preoperative CKD	1.44 (0.19–11.29)	0.727		
Abdominal surgery history	2.36 (0.64–8.79)	0.200		
ACCI score	1.81 (1.36–2.41)	**<0.001**	1.34 (0.54–3.33)	0.533
ECOG PS ≥2	3.77 (2.41–6.93)	**<0.001**	0.40 (0.06–2.56)	0.332
ASA score ≥2	3.05 (0.82–11.32)	0.096		
Preoperative eGFR	0.98 (0.96–1.00)	0.086		
Preoperative hemoglobin	0.97 (0.94–1.00)	0.089		
Laterality				
Left (Re.) vs. Right	0.89 (0.28–2.81)	0.846		
Tumor size	1.29 (0.81–2.06)	0.279		
Clinical T stage ≥cT1b	3.42 (1.03–11.37)	**0.045**	1.42 (0.33–6.18)	0.642
RENAL-NS	1.79 (0.93–3.46)	0.084		
R score	2.75 (0.87–8.68)	0.084		
N score	5.77 (0.03–973.0)	0.503		
L score	1.70 (0.66–4.42)	0.275		
Hilar location	0.62 (0.17–2.29)	0.454		
Technique				
Laparoscopic (Re.) vs. Robot	0.77 (0.23–2.55)	0.665		
Operating method				
PN (Re.) vs. RN	7.36 (1.14–22.99)	**0.038**	7.36 (1.14–47.42)	**0.036**
Postoperative complications	1.02 (0.21–4.99)	0.981		
48-h postoperative AKI	2.72 (0.73–10.06)	0.135		
New onset CKD stage ≥III	1.37 (0.37–5.08)	0.637		
Histologic subtype				
Benign (Re.) vs. Malignant	2.65 (0.34–20.57)	0.351		
Pathologic stage				
pT1 (Re.) vs. pT3	4.24 (1.08–16.66)	**<0.001**	4.24 (1.30–33.21)	**0.039**
Fuhrman grade				
I/II (Re.) vs. III–IV	2.94 (0.58–14.78)	0.191		

BMI, body mass index; ACCI, age-adjusted Charlson's comorbidity index; ECOG PS, Eastern Cooperative Oncology Group Performance Status; ASA, American Society of Anesthesiologists; eGFR, estimated glomerular filtration rate; RENAL-NS, RENAL-Nephrometry Score; RN, radical nephrectomy; PN, partial nephrectomy.

The bold numbers mean statistically difference.

## Discussion

When technically feasible, the management of renal tumors has shifted from RN to PN to reduce the risk of CKD and cardiovascular diseases (6, 7). With the spread of this concept and improvements in surgical technology, the application of PN for renal tumors has been extended to more challenging cases, including ERTs (1). Although several authors have reported their successful experiences and beneficial results (19–21), the evidence in support of PN as a standard is weak. Due to the highly complex branching anatomical structure, it is challenging to remove deep endophytic tumors and suture incised renal parenchyma and hilar structures without increasing perioperative complications; this requires considerable anatomical knowledge and technical skill (2, 9). Park et al. showed that postoperative renal function and contralateral renal volume measured by 3D reconstructive technology according to the endophytic degree of tumors are similar between the OPN and ORN groups. Therefore, they recommended RN as a priority surgical option for ERTs (11). Superior surgical decisions regarding ERTs are of substantial importance; this debate is ongoing. The optimal management of ERTs should balance the potential benefits of intervention with competing risks of mortality in the best interests of these patients. Considering the various risks and benefits of different operating methods, we present here the first report on the perioperative, functional, and oncological outcomes of PN and RN for ERTs.

In this retrospective study, compared with patients who underwent PN, those who underwent RN were more likely to be the elderly with a poor general condition or larger tumor size, and highly complex tumors with high RENAL-NS. Such biases can be identified in other comparative studies between different surgical methods (partial and radical nephrectomy) (22–24). To eliminate selection bias and the influence of confounding factors, PSM analysis was conducted, as shown in **Table 1**. In the matched cohort, patients in the PN group showed a longer OT and more EBL relative to those in the RN group. A systematic review and meta-analysis by Yang et al. comprising 13 retrospective cohort studies with 13,269 patients showed that lower EBL was associated with PN and not RN for T1 renal tumor (25). Li et al. reported a similar result in another meta-analysis (26). Unlike PN, considering the omission in removing tumor and renorrhaphy during RN, the advantages of OT and EBL can be explained. Nevertheless, these advantages do not seem to be conducive to decreasing the length of postoperative hospital stays, which may be related to the similar transfusion rate, conversion rate, recovery time for postoperative bowel function, the duration of drainage, and incidence of overall complications between the two groups.

The occurrence of complications tended to be associated with the anatomical complexity of renal tumors and the basic characteristics of patients (27, 28). No statistically significant differences were observed in the incidence of overall complications between the two groups. However, after categorizing complications by Clavien–Dindo classification, in the intergroup comparison, the PN group was considerably higher than the RN group (10.3% vs. 1.5%, *p* = 0.029). Like in other retrospective studies on PN for the treatment of ERTs (29, 30), high-grade complications included urinary leakage, a requirement for ICU admission, excessive hemorrhage requiring embolization, acute renal failure, and a second operation for persistent drop in blood pressure and hemoglobin. In a meta-analysis involving 30,018 patients with RCC, Yang reported that PN is associated with an increased risk of postoperative hemorrhagic complications and urinary fistula as compared to RN (31). As a complex renal tumor, the risk of complications is higher for ERTs than for general renal tumors. The following surgical difficulties are the reasons for a higher incidence of high-grade complications in the PN group: first, ERTs are generally small in size, deep in location, and have invisible boundaries; thus, it takes a long time to locate and remove the tumor. Furthermore, the resection margin can be positive because of the indistinct tumor expansion during its removal (8), which has a major impact on the prognosis. Second, ERTs are prone to be close to the collecting system and renal sinus, wherein they are highly close to or even infiltrate into the secondary and tertiary renal arteries and veins. It is challenging to remove ERTs with a high risk of accidental vascular or pelvicalyceal system injury, which can lead to accidental rupture, massive bleeding, and urine leakage (7). Finally, even if the kidney tumor is successfully removed, the wound surface is so deep that sewing it up is challenging (9). Because the base of the wound is close to the collecting system or branch blood vessels, inappropriate suture may cause renal vascular occlusion or urine leak from the collecting system. Therefore, we recommend surgeons with rich experience and high technical skills perform PN for ERTs when a tumor-localizing device is available.

Removal of anatomically complicated tumors is inevitably associated with decreased preservation of normal parenchymal nephrons and prolonged WIT, both of which lead to increased renal impairment (32, 33). Despite these adverse factors, our findings showed that patients who underwent PN had less postoperative eGFR reduction (24.3 vs. 35.3 ml/min/1.73 m^2^, *p* = 0.002) and a lower incidence of 48-h postoperative AKI (44.1% vs. 70.6%, *p* = 0.002) relative to those who underwent RN. These results were similar to those of a recent study that investigated the impact factors of perioperative AKI (34). Multivariate analysis showed that the significant predictor of 48-h postoperative AKI was the surgical method, whereby the risk in the RN group was 2.812 times greater than the PN group (*p* = 0.005). In other comparative studies between PN and RN groups, significant differences in perioperative decreased renal function were observed not only for small tumors but also for anatomically complicated tumors (22, 35). The quality and quantity of preserved parenchyma are the main contributors to postoperative long-term renal function (36). The EORTC randomized trial 30904 compared the impact of NSS compared to RN on kidney function in patients with small (≤5 cm) renal tumors. The findings demonstrated that the incidence of at least moderate renal dysfunction was reduced substantially among patients who underwent NSS relative to RN (37). Our results showed similar functional outcomes. PN was more favorable for long-term renal function in patients with ERTs than RN, which was reflected in higher 48-h postoperative eGFR and a higher rate of 1-year postoperative 90% eGFR preservation. At the last follow-up, 20 of 22 cases showed new-onset CKD stage ≥III, wherein the kidney was removed completely. The significant predictors of new-onset CKD stage ≥III were hilar location and RN. It is well known that RN is the main cause of CKD stage ≥III after surgery. Additionally, by calculating the product of eGFR and relative change in renal function before and after surgery for patients with hilar renal tumors, Hinata et al. (38) reported a decrease in the 180-day postoperative renal function; resected weight was the independent predictor of the decrease in the function after PN. This was more likely correlated with preserved normal renal parenchyma and longer WIT due to the location of renal hilar tumors close to the main renal vessels (32, 33, 38).

The survival outcomes before PSM showed that the patients who underwent RN had worse OS relative to those who underwent PN. The loss of functional nephrons is related to a high likelihood of downstream metabolic disorders, including osteoporosis, anemia, and cardiovascular accidents (39, 40). Weight et al. (41) have shown that RN is associated with a 25% increased risk of cardiac death and a 17% increased risk of death due to any cause, ultimately leading to reduced OS (42). Additionally, age, BMI, and ACCI are influencing factors for OS (43–45). After eliminating these influencing factors by PSM, the RN group showed a worse OS status. Multivariate analysis confirmed that RN and pT3 stages were the predictors of overall mortality. The present results showed that non-metastatic pT3 RCC after laparoscopic management incurred metastatic progression of 26% and the three-year mortality rate was 33% (46). A study by Leibovich et al. (47) has shown that the risk ratio for death is 1.87 times higher in patients with peripheral perinephric or renal sinus fat invasion compared with those without fat invasion (p <0.001). Liu et al. (48) compared the survival benefit between PN and RN for renal tumors ≤7 cm with stage pT3a from the SEER database and found that PN yielded better OS for the ≤4 cm group than RN. Furthermore, the Cochrane meta-analysis by Chung et al. (49) compared the oncological outcomes between PN and RN among patients who were upstaged from cT1 renal tumor to pT3a renal cell carcinoma. Relative to RN, patients who underwent PN had better or at least similar oncological outcomes, with a significant improvement in OS, particularly. Therefore, if PN is feasible, RN should be avoided for better survival outcomes among patients with ERTs that can improve in stage.

Interestingly, significant differences between CCS and PFS disappeared after the selection bias and confounding factors of oncological characteristics, including tumor size and RENAL-NS, were balanced by PSM. The results of Palacios et al. (50) indicated that unfavorable oncological outcomes (i.e., CSS and RPS) for localized RCC were mostly associated with aggressive tumor characteristics, not renal function. Zhang et al. (51) reported that tumor size is significantly correlated with nuclear grade and pathological stage, and larger tumors are prone to higher grades and stages. Here, Fuhrman Grade III occurred in 6.9% of renal tumors, which were 2.1 to 4.0 cm in diameter, and 22.3% of tumors between 4.1 and 7 cm in diameter. A study comprising 886 cases of SRM confirmed that increased tumor anatomical complexity quantified by RENAL-NS was independently related to malignancy and high nuclear grade (52). Chen et al. (53) indicated that R- and N-scores were associated with higher postoperative pathological grades. In our study, before PSM, tumors in the RN groups showed larger size (4.1 vs. 2.8 cm, *p* <0.001), higher overall RENAL-NS (10.1 vs. 8.8, *p* <0.001), with higher R score (1.5 vs. 1.2, *p* <0.001), and higher N score (3.0 vs. 2.5, *p* <0.001) relative to the PN group. Thus, all the indicators favorable to tumor progression were skewed toward the RN group. When these significantly different indicators were balanced, the results of similar CSS and PFS in the matched cohort could be easily interpreted.

However, this study is not devoid of limitations. This study was retrospective and based on a single center database. Although PSM analysis was performed to account for the preoperative basic and oncological characteristics, the underlying selection biases or confounding factors may be uncontrollable to a certain extent. Anatomical features and oncological characteristics were assessed by two-dimensional cross-sectional imaging. Therefore, because of the experience and subjective factors of the observers, there might have been judgment biases in this study. Preoperative and postoperative renal functions were not estimated using radioisotope renography, the ideal tool but impractical for every patient. Thus, eGFR was calculated using the internationally recognized Cockgroft–Gault equation, and the results for all patients were standardized by body surface area to improve their reliability. Finally, considering the operating challenges, experienced urologists at a tertiary referral institution performed the procedures, and thus, these results cannot be generalized and should be interpreted with caution.

Despite these limitations, to our knowledge, this retrospective study is the first to evaluate the safety and efficacy of PN and RN for treating ERTs. Additionally, the results of this study are based on PSM analysis to balance influencing factors, which improved the reliability of these findings to a greater extent.

## Conclusion

In conclusion, in the matched cohort of patients with ERTs, RN resulted in more favorable surgical outcomes, but these advantages did not translate into faster postoperative recovery. Additionally, RN was an independent risk factor for short-term and long-term renal function decline. The incidence of overall complications was comparable to that of RN, but PN was more prone to high-grade complications due to the complex anatomical structure of these tumors. Even so, patients who underwent PN showed better preservation of renal function, longer OS, and similar oncological outcomes compared to those who underwent RN. Therefore, we suggest that PN be preferentially considered for ERTs when technically feasible and the surgeon is experienced.

## Data Availability Statement

The raw data supporting the conclusions of this article will be made available by the authors, without undue reservation.

## Ethics Statement

The studies involving human participants were reviewed and approved by Board and the ethical committee of the First Affiliated Hospital of Nanchang University. Written informed consent to participate in this study was provided by the participants’ legal guardian/next of kin. Written informed consent was obtained from the individual(s), and minor(s)’ legal guardian/next of kin, for the publication of any potentially identifiable images or data included in this article.

## Author Contributions

SX and MJ collected the data. SX and BF conceived the manuscript. SX wrote the manuscript. SX and YJ made tables and figures together. WD and BH provides guidance in statistical analysis. RC and ZY provides guidance on the making of figures. LC, XL and XW provided input into the revision of the manuscript. XW sent the manuscript to a native English speaker to correct spelling and grammatical errors. All authors listed have made a substantial, direct, and intellectual contribution to the work and approved it for publication.

## Funding

This study was supported by the National Natural Science Foundation of P.R. China (Grant Nos. 81560419, 81960512, and 81760457), the Jiangxi Provincial “Double Thousand Plan” Fund Project (Grant No. jxsq2019201027), the Key Project of Natural Science Foundation of Jiangxi Province (20212ACB206013), and the Youth Project of Natural Science Foundation of Jiangxi Province (20212BAB216037).

## Conflict of Interest

The authors declare that the research was conducted in the absence of any commercial or financial relationships that could be construed as a potential conflict of interest.

## Publisher’s Note

All claims expressed in this article are solely those of the authors and do not necessarily represent those of their affiliated organizations, or those of the publisher, the editors and the reviewers. Any product that may be evaluated in this article, or claim that may be made by its manufacturer, is not guaranteed or endorsed by the publisher.
